# Longitudinal omics study of transcriptional dynamics in *Lactobacillus crispatus*

**DOI:** 10.1371/journal.pone.0354930

**Published:** 2026-07-30

**Authors:** SiLing Ren, Huimin Jiang, Xiaohua Liu, Daju Zhou, Liping Liu, Lan Wang, Yi Lan, Yi Xiao, Liangdan Tang

**Affiliations:** 1 Department of Obstetrics and Gynecology, The First Affiliated Hospital of Chongqing Medical University, Chongqing, China; 2 Department of Obstetrics and Gynecology, Chongqing Hospital of Jiangsu Province Hospital, Chongqing, China; 3 Department of Obstetrics and Gynecology, Chongqing University Fuling Hospital, Chongqing, China; 4 Department of Obstetrics, Fuling District Maternal and Child Health Hospital, Chongqing, China; 5 Department of Gynecology, Guilin People’s Hospital, Guangxi, China; 6 Department of Gynecology, Women and Children’s Hospital of Chongqing Medical University (Chongqing Health Center for Women and Children), Chongqing, China; 7 Department of Gynecology, Fuling District Maternal and Child Health Hospital, Chongqing, China; Universidade dos Açores Departamento de Biologia: Universidade dos Acores Departamento de Biologia, PORTUGAL

## Abstract

**Background:**

The vaginal microbiome is an important component of female reproductive health. Community State Type I (CST-I), which is dominated by *Lactobacillus crispatus*, is generally considered to be closely associated with a healthy vaginal microecological state. Although the taxonomic composition of CST-I communities remains relatively stable, the transcriptional dynamics of *Lactobacillus crispatus* across different phases of the menstrual cycle remain unclear.

**Methods:**

A healthy reproductive-age woman with a stable CST-I vaginal microbiome was enrolled in this longitudinal study. Vaginal secretion samples were collected at six non-menstrual time points throughout a menstrual cycle. Third-generation full-length 16S rRNA gene sequencing and metatranscriptomic sequencing were performed. Differential transcript expression analysis, KEGG pathway enrichment analysis, and Mfuzz time-series clustering were applied to systematically characterize the transcriptional dynamics of the vaginal microbiome across different phases.

**Results:**

Full-length 16S rRNA gene sequencing confirmed that *Lactobacillus crispatus* remained the dominant species at all six sampling time points, with no substantial changes in overall community composition. However, metatranscriptomic analysis revealed pronounced phase-specific transcriptional reprogramming. During the pre-ovulatory phase, pathways involved in DNA replication, biosynthesis, and central carbon metabolism were upregulated. On the first day after ovulation, pathways associated with carbohydrate utilization, quorum sensing, and redox homeostasis were selectively upregulated. During the mid-to-late luteal phase, pathways related to cell proliferation and growth were generally downregulated, whereas pathways involved in cofactor biosynthesis, the pentose phosphate pathway, and D-amino acid metabolism showed increased expression. Mfuzz clustering further revealed distinct phase-specific functional transitions in *L. crispatus* that were absent in non-*L. crispatus* lactobacilli.

**Conclusion:**

The ecological dominance of *Lactobacillus crispatus* is maintained not solely through numerical abundance, but also through rhythmic and phase-specific transcriptional regulation. Dynamic functional remodeling and metabolic plasticity may represent important mechanisms underlying its long-term persistence and ecological dominance within healthy CST-I vaginal communities.

## 1. Introduction

The vaginal microbiome is a fundamental component of women’s reproductive health. A healthy vaginal microbial community structure and functional activity are essential for maintaining a low vaginal pH, preventing colonization by exogenous pathogens, and reducing the risk of gynecological disorders. In healthy women, vaginal microecological balance is primarily maintained by *Lactobacillus* species, which ferment glycogen into lactic acid and thereby sustain an acidic vaginal environment (pH 3.8–4.5). In addition, *Lactobacillus* species produce antimicrobial substances, including hydrogen peroxide and bacteriocins, which inhibit the overgrowth of pathogenic microorganisms [1].

Based on 16S rRNA gene sequencing, the vaginal microbiome can be classified into five Community State Types (CSTs): CST-I is dominated by *Lactobacillus crispatus*, CST-II by *Lactobacillus gasseri*, CST-III by *Lactobacillus iners*, and CST-V by *Lactobacillus jensenii*, whereas CST-IV is characterized by a diverse assemblage of anaerobic bacteria accompanied by a marked reduction in *Lactobacillus* abundance [2]. Among these community state types, CST-I has been most consistently linked with a healthy vaginal condition [2].

However, the menstrual cycle induces dynamic changes in the vaginal microenvironment, presenting phase-specific challenges to the survival and metabolic strategies of vaginal *Lactobacillus* species [3]. During the follicular phase, rising estrogen levels stimulate glycogen accumulation in vaginal epithelial cells, while cervical secretions become thinner and more hydrated, creating a nutrient-rich environment that supports carbohydrate metabolism by *Lactobacillus* species and facilitates maintenance of vaginal acidity [4–5]. In the early luteal phase, rapidly elevated progesterone levels antagonize estrogen-induced vaginal epithelial proliferation and drive superficial epithelial cells toward terminal differentiation and programmed shedding [6]. Concurrently, cervical mucus becomes more viscous, restricting nutrient diffusion, while transient neutrophil infiltration may contribute to a temporary increase in oxidative stress within the vaginal environment [7]. During the mid-to-late luteal phase, sustained progesterone exposure suppresses epithelial proliferation and limits glycogen replenishment [8]. Consequently, the vaginal ecosystem gradually shifts toward a state characterized by nutrient depletion and accumulation of oxidative metabolites, further challenging the persistence of resident microbial communities.

Previous studies have verified that the menstrual cycle modulates vaginal microbiome composition, yet the molecular mechanisms whereby vaginal microbes adapt to microenvironmental fluctuations via transcriptional regulation remain unclear [9]. In this study, one healthy woman with a stable vaginal microbiome classified as CST I was enrolled. By combining third-generation full-length 16S rRNA sequencing and metatranscriptomics of vaginal swabs collected at six time points, we systematically characterized phase-specific functional features and environmental adaptation strategies of the dominant species *Lactobacillus crispatus*.

## 2. Materials and Methods

### 2.1. Volunteer recruitment and sample collection

This study is part of a larger vaginal microbiome transplantation research program based on *Lactobacillus crispatus*.To identify a representative individual with a CST-I vaginal microbiome for longitudinal multi-omics analysis across the non-menstrual phases of the menstrual cycle, healthy reproductive-age women were recruited through offline advertisements between September 1, 2024 and December 31, 2024. The inclusion criteria were age between 18–40 years, regular menstrual cycles, and absence of self-reported symptoms related to vaginal inflammation. The exclusion criteria included use of antibiotics or probiotics within the previous 3 months, a history of vaginal douching within the past month, current pregnancy or breastfeeding, irregular menstrual cycles, the presence of any chronic underlying diseases or immunocompromising conditions, and detection of vaginal pathogenic microorganism colonization or infection.

All participants first underwent preliminary screening through questionnaires administered by medical staff from the research team. Participants who met the above inclusion and exclusion criteria subsequently underwent vaginal microecological evaluation. Vaginal microecological evaluation was performed using Gram-stained vaginal secretion smears combined with routine vaginal microecological functional indicators. Morphological evaluation included epithelial cell quantity, bacterial density, bacterial diversity, dominant bacterial type, Nugent score, and Aerobic Vaginitis score, together with microscopic observation for the presence of clue cells, *Mobiluncus*, Gram-positive cocci, Gram-negative rods, yeast spores, yeast hyphae, *Trichomonas vaginalis*, and Gram-negative diplococci or other abnormal microbial morphotypes. Functional evaluation included vaginal pH and qualitative detection of hydrogen peroxide (H_2_O_2_), leukocyte esterase (LE), and BV-associated sialidase [10].

Vaginal microecological balance was defined as a *Lactobacillus*-dominant microbiome (*Lactobacillus* relative abundance >75%), Nugent score of 0–3, Aerobic Vaginitis score of 0–3, vaginal pH maintained between 3.8 and 4.5, positive hydrogen peroxide activity, and negative leukocyte esterase activity [11]. In addition, Gram-stained microscopic examination of vaginal secretion smears showed no clue cells, fungal spores or hyphae, *Trichomonas, Neisseria gonorrhoeae,* or other definitive pathogenic microbial morphotypes [11]. Individuals who met the above inclusion and exclusion criteria and exhibited characteristics consistent with vaginal microecological balance were considered eligible for subsequent third-generation full-length 16S rRNA gene sequencing analysis.

Among all participants who underwent third-generation full-length 16S rRNA gene sequencing analysis, the participant with the highest relative abundance of *Lactobacillus crispatus* in the vaginal microbiome was selected as the final study participant. To perform longitudinal multi-omics analysis of the vaginal microbiome during the non-menstrual phases of the menstrual cycle, vaginal secretion samples were collected at six different time points after menstruation had ceased. To determine the ovulation day, a combined strategy integrating basal body temperature monitoring and menstrual cycle prediction was adopted for comprehensive assessment.

All samples were collected by gynecologists with more than five years of clinical experience in an outpatient examination room using sterile single-use vaginal swabs (Kangjie; Jiangsu Kangjie Medical Equipment Co., Ltd., Jiangsu, China). During collection, a sterile speculum was used to expose the posterior vaginal fornix, and swabs were used to collect leucorrhea secretions from the posterior fornix. At each sampling time point, at least six vaginal swabs were collected. After collection, the front end of each swab was aseptically cut using sterile scissors and immediately transferred into sterile cryovials (Corning Cryogenic Vials, Corning Inc., USA). The samples were aliquoted into three cryovials, with two leucorrhea swabs stored per cryovial. All samples were immediately stored at −80 °C until further analysis.

### 2.2. Full-Length 16S rRNA gene sequencing analysis

#### 2.2.1. Sample pretreatment and DNA extraction.

Total genomic DNA was extracted from vaginal secretion swab samples using the TGuide S96 Magnetic Beads Genomic DNA Extraction Kit (Tiangen Biotech, China). Swabs stored at −80 °C were rapidly thawed and immersed in 2 mL microcentrifuge tubes containing 500 μL Buffer SA and 100 μL Buffer SC. After thorough elution, the swab shafts were removed and discarded. The eluates were transferred to bead-beating tubes preloaded with 0.25 g grinding beads and mechanically disrupted using a TGrinder H24 tissue homogenizer (OSE-TH-01) at 6 m/s for two cycles of 30 s each, with a 30 s interval between cycles, to enhance cell wall disruption. The homogenates were incubated at 70 °C for 15 min to ensure complete cell lysis, followed by centrifugation at 12,000 rpm for 1 min. Approximately 500 μL of supernatant was collected.

Subsequently, 200 μL Buffer SH was added to the supernatant and mixed thoroughly. After incubation at 4 °C for 10 min, the mixture was centrifuged again at 12,000 rpm for 3 min. The clarified supernatant was transferred to a new tube, mixed with 500 μL Buffer GFA pre-mixed with an equal volume of isopropanol, and inverted. Ten microliters of magnetic bead suspension (Buffer G) was added, followed by continuous shaking at 200 rpm for 5 min at room temperature to facilitate DNA binding. The mixture was placed on a magnetic rack for 30 s, and the supernatant was discarded.

The beads were sequentially washed with 700 μL protein removal buffer RD (pre-mixed with absolute ethanol) and twice with 700 μL wash buffer PWD (containing absolute ethanol). After each wash, the beads were resuspended by gentle mixing and magnetically separated to remove the supernatant. The beads were then air-dried on the magnetic rack for 5–10 min. Finally, DNA was eluted in 50 μL elution buffer TB by incubation at 56 °C for 5 min with intermittent mixing.

#### 2.2.2. DNA quality assessment and full-length 16S rRNA gene amplification.

DNA concentration was further quantified using a Qubit 4.0 fluorometer with the Qubit™ dsDNA HS Assay Kit to ensure a concentration of ≥10 ng/μL. DNA integrity was evaluated by 1.0% agarose gel electrophoresis (GelRed staining, 100 V for 30 min; Bio-Rad, USA).

The full-length 16S rRNA gene (V1–V9 regions) was amplified using the universal primer pair 27F (5′-AGRGTTTGATYNTGGCTCAG-3′) and 1492R (5′-TASGGHTACCTTGTTASGACTT-3′) [12]. PCR reactions were performed in a total volume of 50 μL, containing 25 μL of 2 × Phanta Max Master Mix (Vazyme, China), 1 μL of each primer stock solution (10 μM; final concentration, 0.2 μM each), 50 ng template DNA, and nuclease-free water to volume. Thermal cycling conditions were as follows: initial denaturation at 95 °C for 3 min; 30 cycles of denaturation at 95 °C for 15 s, annealing at 55 °C for 15 s, and extension at 72 °C for 60 s; followed by a final extension at 72 °C for 5 min.

#### 2.2.3. PCR Product Validation and PacBio Library Construction.

PCR products were examined by 1.8% agarose gel electrophoresis (GelRed staining, 5 V/cm for 30 min; Bio-Rad, USA). Amplicons showing a dominant band of approximately 1,500 bp without obvious nonspecific amplification were quantified using the LabChip GX Touch system and pooled in equimolar amounts. SMRTbell libraries were prepared using the PacBio SMRTbell Prep Kit 3.0 (PacBio, USA) according to the manufacturer’s instructions and sequenced on the PacBio Revio platform.

#### 2.2.4. Bioinformatics Analysis of Full-Length 16S rRNA Gene Sequencing Data.

Raw subreads were corrected using SMRT Link v11.0 to generate circular consensus sequencing (CCS) reads. Primer sequences were identified and removed using cutadapt v1.8.3, and reads outside the length range of 1,200–1,650 bp were filtered out. Chimeric sequences were identified and removed using the UCHIME algorithm. High-quality non-chimeric reads were clustered into operational taxonomic units (OTUs) at 97% sequence similarity using VSEARCH v2.4.3. Taxonomic annotation was performed using BLASTn against the SILVA database (Release 138) combined with the Lowest Common Ancestor (LCA) algorithm, with unassigned sequences further complemented by the naive Bayesian classifier implemented in QIIME2.

### 2.3. Metatranscriptomic Sequencing and Bioinformatic Analysis

#### 2.3.1. RNA Extraction and Quality Assessment.

Vaginal secretion samples were retrieved from −80 °C storage and immediately subjected to RNA extraction. RNase-free PBS buffer was added to the cryovial containing the swab tip. The sample was vortexed to elute cells from the swab, after which the swab was removed and discarded. The cell suspension was transferred to a new RNase-free tube and centrifuged at 5,000 × g for 5 min at 4 °C to pellet the cells. The supernatant was discarded, and 1 mL of TRIzol reagent was added directly to the cell pellet, followed by vigorous vortexing for 30 s to ensure complete cell lysis. Following the addition of 200 μL of chloroform, the mixture was shaken vigorously for 15 s and incubated at room temperature for 3 min. After centrifugation at 12,000 × g for 15 min at 4 °C, the aqueous phase was carefully transferred to a new RNase-free tube. RNA was precipitated by adding an equal volume of isopropanol and incubating at −20 °C for 1 h. The RNA pellet was collected by centrifugation at 12,000 × g for 10 min at 4 °C, washed twice with 75% ethanol (prepared with RNase-free water), air-dried for 5–10 min, and dissolved in 30 μL of RNase-free water.

RNA quality was assessed using a multi-step approach. RNA degradation and potential contamination were preliminarily evaluated by 1.0% agarose gel electrophoresis (GelRed staining, 5 V/cm for 15 min; Bio-Rad, USA). RNA concentration and purity were measured using a NanoDrop One spectrophotometer. RNA integrity was further evaluated using an Agilent 5300 Fragment Analyzer (Agilent Technologies, Santa Clara, CA, USA), and the RNA Quality Number (RQN) was recorded. According to the quality control criteria, RNA samples with RQN ≥ 4.5, a minimum input of 1 μg total RNA for library construction, and no obvious pigment, protein, or carbohydrate contamination were considered qualified and used for subsequent metatranscriptomic library preparation.

#### 2.3.2. Library preparation and sequencing.

Qualified RNA samples were treated with the Ribo-Zero rRNA Removal Kit (Illumina, USA) to deplete ribosomal RNA and enrich messenger RNA (mRNA). The rRNA-depleted RNA was subjected to heat-induced random fragmentation in the fragmentation buffer supplied with the kit. Briefly, the reaction mixture was incubated at 94 °C for 8 min to generate randomly fragmented RNA with an average size suitable for Illumina library construction (approximately 200–300 nt). The fragmentation reaction was immediately terminated by placing the samples on ice.

First-strand cDNA synthesis was then performed using random hexamer primers and SuperScript II reverse transcriptase (Thermo Fisher Scientific, USA, 18064014), followed by second-strand cDNA synthesis using DNA Polymerase I (New England Biolabs, USA, M0209S) and RNase H (New England Biolabs, USA, M0297S). The resulting double-stranded cDNA was purified using AMPure XP magnetic beads (Beckman Coulter, USA), followed by end repair, 3′ adenylation, and adaptor ligation using the VAHTS Universal DNA Library Prep Kit for Illumina V3 (Vazyme, China) according to the manufacturer’s instructions. Adaptor-ligated products were size-selected for fragments of approximately 200–400 bp using magnetic bead-based purification. The selected libraries were subsequently enriched by PCR amplification using library-specific primers. PCR amplification consisted of an initial denaturation at 98 °C for 30 s, followed by 15 cycles of 98 °C for 10 s, 60 °C for 30 s, and 72 °C for 20 s, with a final extension at 72 °C for 5 min. The amplified products were purified using AMPure XP magnetic beads (Beckman Coulter, USA) to generate the final sequencing libraries. Library concentration and fragment size distribution were assessed using Qubit 2.0 and the Agilent 2100 Bioanalyzer. Libraries with concentrations greater than 2 nM were subjected to paired-end sequencing on an Illumina HiSeq platform using the Illumina HiSeq PE Cluster Kit and HiSeq SBS Kit (Illumina, USA) according to the manufacturer’s instructions.

#### 2.3.3. Quality Control and Transcriptome Assembly.

Raw sequencing reads were subjected to stringent quality control procedures based on FastQC criteria for Illumina sequencing data. Low-quality reads were defined as reads in which more than 50% of bases had Phred quality scores ≤20. High-quality clean reads were retained for downstream analyses. Sequencing quality was evaluated using FASTQC to calculate Q20/Q30 scores, GC content, and base quality distributions to confirm random fragmentation patterns and overall sequencing reliability.

Clean reads were aligned to the NCBI rRNA database using Bowtie2 to remove residual rRNA-derived reads. Unmapped reads were considered non-rRNA (mRNA) reads and were assembled de novo using MEGAHIT [13]. Transcripts assembled from individual samples were merged and clustered using CD-HIT-EST with a sequence identity threshold of 95% to generate a non-redundant unigene set. Assembly quality was assessed by calculating total transcript number, total length, average length, N50 value, and maximum transcript length.

#### 2.3.4. Taxonomic and Functional Annotation.

Representative unigene sequences were aligned against the NCBI non-redundant protein database (NR) using DIAMOND BLASTx with an E-value cutoff of ≤ 1 × 10 ^−^ ^5^. Taxonomic annotation was performed using the Lowest Common Ancestor (LCA) algorithm implemented in MEGAN [14]. For functional annotation, translated unigene sequences were searched against the KEGG database using DIAMOND BLASTx, with an E-value threshold of ≤ 1 × 10 ^−^ ^5^ and minimum query and reference sequence coverage of 40%. Functional pathways were parsed based on KEGG Pathway annotations.

## 3. Statistical methods

All statistical analyses were performed using R software (version 4.3.3). For vaginal microbiome composition analysis, based on 16S rRNA gene sequencing data, alpha diversity indices, including observed features, ACE richness, Shannon diversity, and Faith’s phylogenetic diversity (PD), were calculated from rarefied feature tables. Differences in alpha diversity among menstrual cycle phases were assessed using the Kruskal-Wallis test. Beta diversity was calculated using Bray-Curtis, Jaccard, unweighted UniFrac, and weighted UniFrac distance matrices, and differences in microbial community composition across menstrual cycle phases were evaluated using permutational multivariate analysis of variance (PERMANOVA, Adonis). For metatranscriptomic analysis, transcript abundance was normalized to transcripts per million (TPM) for expression profiling and visualization. Differential expression analysis was performed using DESeq2, and differentially expressed transcripts were identified using thresholds of |log_2_ fold change| > 1 and a false discovery rate (FDR) < 0.05. Differentially expressed transcripts were visualized using MA plots. Functional enrichment analysis of differentially expressed transcripts was conducted based on KEGG pathway annotations, and pathway-level transcriptional changes were summarized at the species level to characterize species-specific functional dynamics across different phases of the menstrual cycle.

## 4. Results

### 4.1. Participant Screening and Identification of a Representative CST-I Individual

A total of 18 reproductive-age women completed baseline questionnaires in this study. Based on the questionnaire screening results, six participants failed to meet the inclusion criteria, including two with recent antibiotic use, one with irregular menstrual cycles, one with a history of vaginal douching, and two with chronic underlying diseases. In addition, two participants withdrew from the study before subsequent procedures were performed. Ultimately, ten participants proceeded to the vaginal microecological evaluation phase.

During the vaginal microecological evaluation, one participant was diagnosed with bacterial vaginosis (BV), and four participants were excluded from subsequent analysis due to vaginal microecological imbalance. Among these four, two participants had elevated Nugent scores, and the other two exhibited a relative abundance of *Lactobacillus* below 75%. Ultimately, five participants met the criteria for vaginal microecological balance.

**Fig 1:**
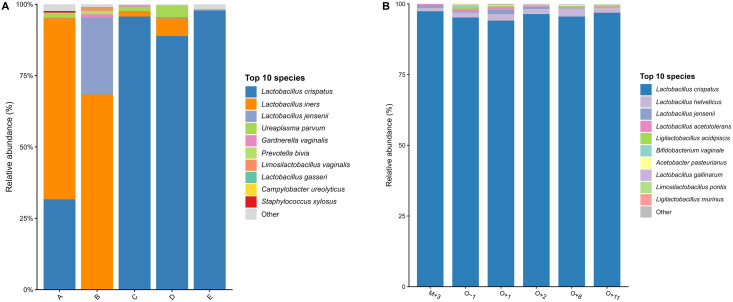
1A. Species-level vaginal microbiome composition of the five participants based on 16S rRNA gene sequencing. **1B. Species-level vaginal microbiome composition of participant E across six non-menstrual time points during the menstrual cycle.** Note: Stacked bar plots illustrate the relative abundance (%) of bacterial species in vaginal samples from the five participants. All participants exhibited *Lactobacillus*-dominated microbial communities, with *Lactobacillus* accounting for more than 75% of the total relative abundance. Participants A and B were predominantly dominated by *Lactobacillus iners*, whereas participants C, D, and E exhibited communities dominated by *Lactobacillus crispatus*. Participant E showed the lowest relative abundance of non-*Lactobacillus* taxa among the five individuals.Other represents the sum of relative abundances of all bacterial taxa not shown separately in the figure. Note: Stacked bar plots show the relative abundance (%) of bacterial species in vaginal samples from participant E at six non-menstrual time points across the menstrual cycle (M + 3, O − 1, O + 1, O + 2, O + 8, and O + 11).

Subsequently, vaginal secretion samples from the above five participants were subjected to third-generation full-length 16S rRNA gene sequencing analysis (Fig 1A). The results showed that the vaginal microbiome of all five participants were dominated by *Lactobacillus* species. Participants A and B were primarily dominated by *Lactobacillus iners*, whereas Participants C, D, and E were predominantly dominated by *Lactobacillus crispatus*. Participant E was selected as the final participant for subsequent longitudinal multi-omics analysis, as she exhibited the highest relative abundance of *Lactobacillus crispatus* and was negative for *Ureaplasma parvum*.

### 4.2. Definition of Vaginal Swab Collection Time Points and Corresponding Menstrual Cycle Phases in Participant E

**Fig 2:**
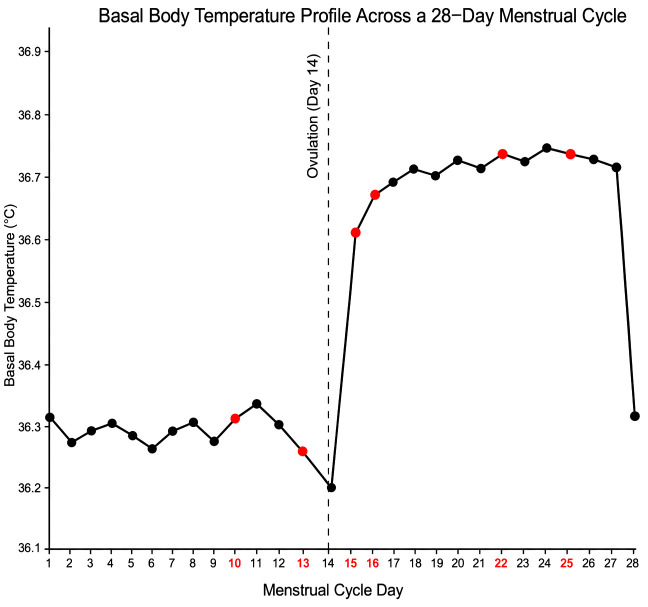
Basal body temperature profile of participant E across a 28-day menstrual cycle. Note: Daily basal body temperature (BBT) measurements in participant E demonstrated a typical biphasic pattern throughout the menstrual cycle. Starting from the first day of menstruation, BBT was measured each morning (7:30–8:00 a.m.) while the participant remained at rest in bed, using a mercury thermometer (Dong’e Ahua Medical Technology Co., Ltd.). Ovulation was determined based on two criteria: (1) a sustained increase in BBT of at least 0.3 °C for more than three consecutive days following the lowest temperature point, and (2) calendar-based estimation placing ovulation 14 days before the next expected menstruation. The day with the lowest BBT before the sustained temperature rise was defined as the ovulation day (Day 14). The dashed line indicates the estimated ovulation day. Red dots indicate the sampling time points.

Participant E had a regular 28-day menstrual cycle with a menstrual duration of 7 days. Vaginal secretion samples were collected on days 10, 13, 15, 16, 22, and 25 of the menstrual cycle. Based on retrospective ovulation determination using basal body temperature monitoring, these six sampling time points were subsequently defined as the third day after menstruation (M + 3), one day before ovulation (O − 1), the first day after ovulation (O + 1), the second day after ovulation (O + 2), the eighth day after ovulation (O + 8), and the eleventh day after ovulation (O + 11) (Fig 2). According to different menstrual cycle phases, the six sampling time points were further classified into Phase A (M + 3 and O − 1), Phase B (O + 1 and O + 2), and Phase C (O + 8 and O + 11), representing the follicular phase, early luteal phase, and mid-to-late luteal phase, respectively. Further analysis of the vaginal microbiome composition of Participant E at six time points revealed that her vaginal microbiome remained consistently dominated by *Lactobacillus crispatus* across all three phases of the sampling period (Fig 1B).

### 4.3. Alpha and Beta Diversity Analysis of the Vaginal Microbiome in Participant E During Non-Menstrual Phases

**Fig 3:**
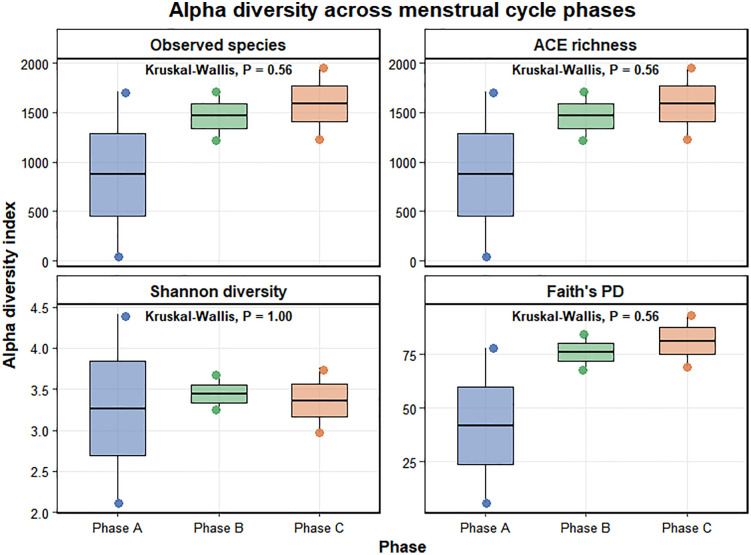
Alpha diversity of the vaginal microbiome in participant E across three phases. Note: Alpha diversity was evaluated using four indices: observed species richness, ACE richness index, Shannon diversity index, and Faith’s phylogenetic diversity (PD). Samples were phased as follows: Phase A included M + 3 and O − 1, Phase B included O + 1 and O + 2, and Phase C included O + 8 and O + 11. Differences among phases were assessed using the Kruskal-Wallis test, with P < 0.05 considered statistically significant.

To assess whether the richness and evenness of the vaginal microbiome in vaginal secretion samples from Participant E varied across the phases, we first compared alpha diversity indices among the three phases. Alpha diversity analyses revealed no statistically significant differences among the three phases across all evaluated indices (Fig 3; Observed species: P = 0.56; ACE richness: P = 0.56; Shannon diversity: P = 1.00; Faith’s PD: P = 0.56). Although Phase A showed a relatively broader distribution range in observed species richness and ACE richness compared with Phases B and C in Fig 3, Kruskal-Wallis tests did not support statistically significant differences among phases.

**Fig 4:**
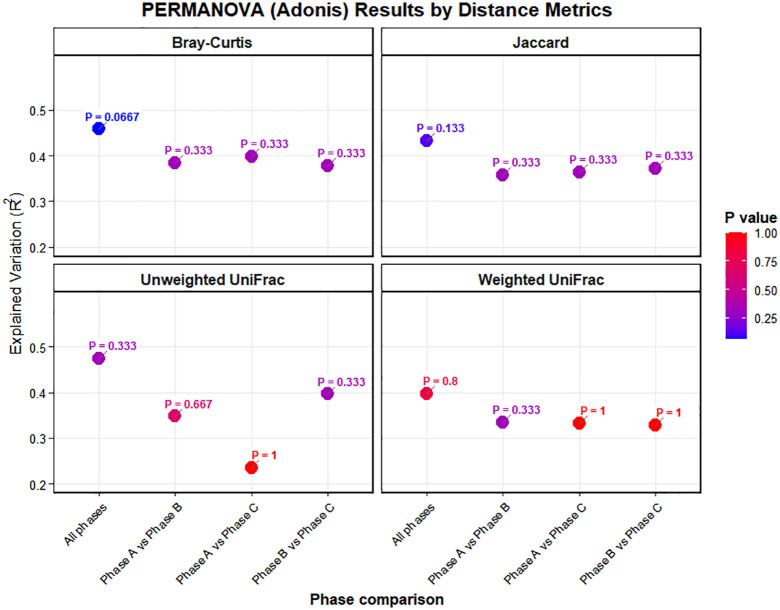
PERMANOVA analysis of the vaginal microbiome in participant E across three phases. Note: Permutational multivariate analysis of variance (PERMANOVA) was performed to evaluate differences in vaginal microbiome community structure among three phases in participant E. Analyses were conducted based on four distance metrics, including Bray-Curtis, Jaccard, unweighted UniFrac, and weighted UniFrac distances. Statistical significance was defined as P < 0.05.

Subsequently, we performed beta diversity analysis to explore whether the overall compositional structure of the vaginal microbial community differed among the three phases (Fig 4). In the overall comparison across all phases, Bray-Curtis distances explained a relatively higher proportion of variance (R^2^ = 0.458, P = 0.067), although this did not reach statistical significance. No significant differences were observed for the other distance metrics (Jaccard: R^2^ = 0.433, P = 0.133; unweighted UniFrac: R^2^ = 0.475, P = 0.333; weighted UniFrac: R^2^ = 0.398, P = 0.800). Pairwise comparisons between phases (Phase A vs. Phase B, Phase A vs. Phase C, and Phase B vs. Phase C) likewise revealed no statistically significant differences across all distance measures (all adjusted P values > 0.05) (Fig 4).

### 4.4. Differential transcript expression profiles of the vaginal microbiome in participant E Across three phases

**Fig 5:**
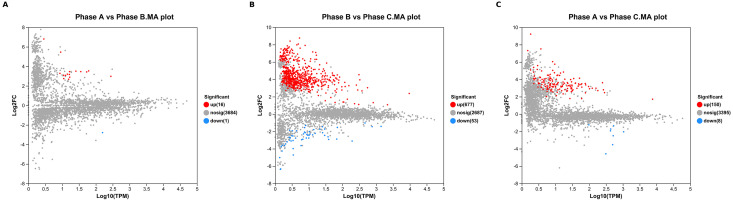
Differentially expressed transcripts of the Vaginal Microbiome in Participant E Across Three Phases Based on Metatranscriptomic Analysis. Note: The MA plot illustrates the differential transcript expression profiles across three phases. Upregulated transcripts are shown in red, downregulated transcripts in blue, and non-significant transcripts are shown in grey. “Upregulated” indicates higher expression levels in the former phase relative to the latter phase in each pairwise comparison, whereas “downregulated” indicates the opposite.

To investigate the functional transcript expression changes of the vaginal microbiome in Participant E, we performed pairwise comparisons of metatranscriptomic profiles across three phases. In the Phase A vs. Phase B comparison, a total of 3,701 transcripts were included in the analysis, among which 17 differentially expressed transcripts (DETs) were identified, including 16 upregulated and 1 downregulated transcripts, while 3,684 transcripts did not reach statistical significance (Fig 5A). In the Phase B vs. Phase C comparison, a total of 3,417 transcripts were included in the analysis, among which 730 differentially expressed transcripts were identified, including 677 upregulated and 53 downregulated transcripts, while 2,687 transcripts did not reach statistical significance (Fig 5B). In the Phase A vs. Phase C comparison, a total of 3,553 transcripts were included in the analysis, among which 158 differentially expressed transcripts were identified, including 150 upregulated and 8 downregulated transcripts, while 3,395 transcripts did not reach statistical significance (Fig 5C).

### 4.5. KEGG Pathway Enrichment Analysis of Differentially Expressed Transcripts in Participant E

**Fig 6:**
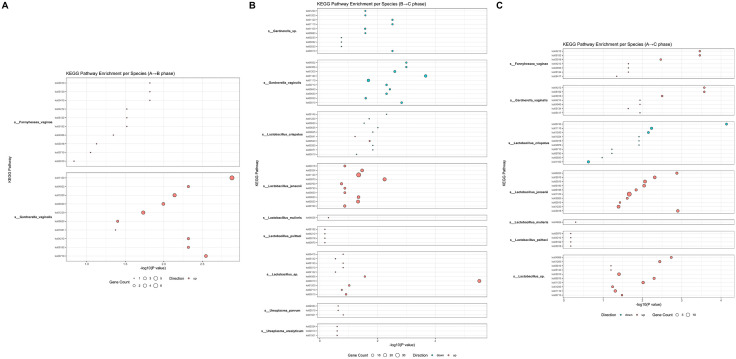
KEGG pathway enrichment analysis of differentially expressed transcripts in participant E. Note: Bubble plots display KEGG pathway enrichment results for differentially expressed transcripts. Enrichment analyses were performed in a species-resolved manner. “Upregulated” indicates higher expression levels in the former phase relative to the latter phase, whereas “Downregulated” indicates lower expression levels in the former phase. (A) Comparison between Phase A (M + 3 and O − 1) and Phase B (O + 1 and O + 2). (B) Comparison between Phase B (O + 1 and O + 2) and Phase C (O + 8 and O + 11). (C) Comparison between Phase A (M + 3 and O − 1) and Phase C (O + 8 and O + 11).

To further characterize the biological pathways underlying the observed transcriptional changes, we performed KEGG pathway enrichment analysis on the differentially expressed transcripts in a species-resolved manner. In the Phase A vs. Phase B comparison, differentially expressed transcripts were mainly annotated to potentially pathogenic taxa, whereas no significant differential transcriptional changes were detected in *Lactobacillus crispatus*. During Phase B, the downregulated pathways associated with *Gardnerella vaginalis* were mainly involved in central carbon metabolism and biosynthetic processes, including pyruvate metabolism (ko00620), glycolysis/gluconeogenesis (ko00010), the pentose phosphate pathway (ko00030), amino sugar and nucleotide sugar metabolism (ko00520), and one-carbon pool by folate (ko00670) (Fig 6A), whereas the downregulated pathways associated with *Fannyhessea vaginae* were mainly related to host interaction and virulence-associated functional modules, including bacterial invasion of epithelial cells (ko05152), pathogenic *Escherichia coli* infection (ko05130), and shigellosis (ko05131) (Fig 6A).

During the transition from Phase B to Phase C, differentially expressed transcripts of *Lactobacillus crispatus* were predominantly enriched in energy metabolism–related pathways, with significant upregulation of fatty acid metabolism (ko00625, ko00626), butanoate metabolism (ko00650), pentose and glucuronate interconversions (ko00071), aminoacyl-tRNA biosynthesis (ko00350), and global metabolic pathways (ko01220) in Phase C (Fig 6B). Compared with Phase B, *Gardnerella vaginalis* showed marked upregulation of several metabolism-associated pathways in Phase C, mainly involving carbon metabolism (ko01200), microbial metabolism in diverse environments (ko01120), biosynthesis of secondary metabolites (ko01110), metabolic pathways (ko01100), as well as energy and intermediate metabolism–related pathways, including ko00710, ko00640, ko00620, and ko00010 (Fig 6B). In contrast, *Lactobacillus jensenii* exhibited predominant downregulation of pathways associated with energy and material metabolism in Phase C compared with Phase B, including oxidative phosphorylation, pyruvate metabolism, butanoate metabolism, and pathways related to vitamin and cofactor metabolism. Meanwhile, *Lactobacillus* sp. (unclassified *Lactobacillus* species) also showed reduced expression of multiple pathways involved in carbon metabolism, energy metabolism, and metabolic integration during Phase C (Fig 6B).

Compared with Phase A, *Lactobacillus crispatus* exhibited significant upregulation of multiple pathways during Phase C, including riboflavin metabolism (ko00740), biosynthesis of cofactors (ko01240), biosynthesis of secondary metabolites (ko01110), the MAPK signaling pathway (ko04016), and the platinum drug resistance pathway (ko01524) (Fig 6C). In contrast, both *Fannyhessea vaginae* and *Gardnerella vaginalis* displayed significant downregulation of multiple functional pathways during Phase C relative to Phase A. The downregulated pathways commonly involved ABC transporters (ko02010), biosynthesis of secondary metabolites (ko01110), ribosome-related pathways (ko03010), the MAPK signaling pathway (ko04212), platinum drug resistance pathway (ko05152), bile secretion (ko04940), amoebiasis (ko05134), and parasitic infection-related pathways (ko05417) (Fig 6C).

### 4.6. Mfuzz Soft Clustering Analysis of Vaginal Microbial Transcriptional Dynamics in Participant E

To characterize time-resolved transcriptional dynamics of key vaginal bacteria across the non-menstrual phases of the menstrual cycle, Mfuzz soft clustering was applied to metatranscriptomic time-series data from *Lactobacillus crispatus*, non-*L. crispatus* lactobacilli, and *Gardnerella vaginalis*. The time-series clustering analysis in Mfuzz provided a visual representation of dynamic transcriptional trajectories, phasing transcripts with similar temporal expression patterns into the same cluster.

**Fig 7:**
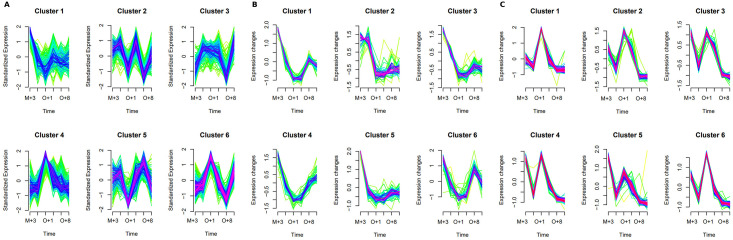
Mfuzz-based dynamic cluster analysis of time-series metatranscriptomic data from the vaginal microbiome of participant E. Note: Mfuzz soft clustering was applied to time-series metatranscriptomic data to identify dynamic transcript expression clusters across six time points. Panel A shows the clustering results for *Lactobacillus crispatus*, Panel B for *Gardnerella vaginalis*, and Panel C for non-*L. crispatus* lactobacilli.

**Fig 8:**
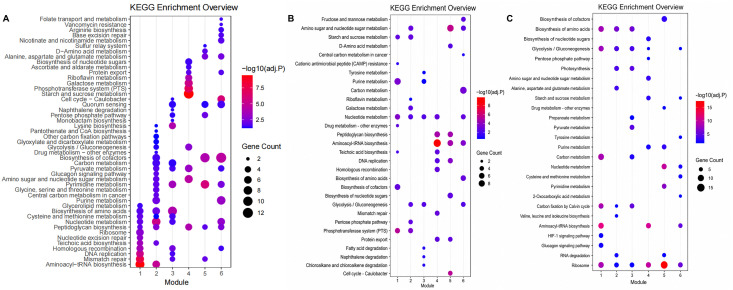
KEGG Functional Enrichment of Mfuzz Transcriptional Clusters in the Vaginal Microbiome of Participant E. Note: Bubble plots show KEGG pathway enrichment results for six transcriptional clusters generated by Mfuzz soft clustering analysis. The x-axis represents different Mfuzz clusters, and the y-axis represents enriched KEGG pathways. Bubble size indicates the number of transcripts enriched in each pathway, while bubble color represents the enrichment significance level [−log10(adjusted P value)]. Panel A represents *Lactobacillus crispatus*, Panel B represents *Gardnerella vaginalis*, and Panel C represents non-*L. crispatus* lactobacilli.

Temporal clustering analysis of *Lactobacillus crispatus* transcripts identified six distinct expression trajectories (Fig 7A). Among these, clusters 1 and 2 displayed a broadly similar temporal pattern characterized by relatively elevated expression during the early cycle phase (M + 3 and O − 1), followed by marked suppression at O + 1 and subsequent partial recovery during the mid-to-late luteal phase. Functional enrichment analysis suggested that these clusters were primarily associated with core cellular maintenance and biosynthetic activities. Specifically, cluster 1 was enriched in pathways related to DNA replication and repair, including mismatch repair, nucleotide excision repair, homologous recombination, and DNA replication, as well as pathways involved in ribosome biogenesis, peptidoglycan biosynthesis, teichoic acid biosynthesis, and amino acid biosynthesis. Cluster 2 was primarily associated with nucleotide and energy metabolism, including purine metabolism, pyrimidine metabolism, glycolysis/gluconeogenesis, pyruvate metabolism, and carbon metabolism (Fig 8A).

In contrast, clusters 3, 4, and 6 exhibited an ovulation-associated activation pattern, with transcriptional upregulation predominantly observed at O + 1 (Fig 7A). Cluster 4 was mainly enriched in pathways related to carbohydrate utilization and transport, including starch and sucrose metabolism, phosphotransferase system (PTS), galactose metabolism, nucleotide sugar biosynthesis, and amino sugar and nucleotide sugar metabolism. Cluster 6 was enriched in pathways associated with *Caulobacter* cell cycle regulation, nicotinate and nicotinamide metabolism, base excision repair, arginine biosynthesis, as well as purine and nucleotide metabolism. Meanwhile, cluster 3 was associated with lysine biosynthesis, amino acid biosynthesis, pyrimidine metabolism, homologous recombination, quorum sensing, and *Caulobacter* cell cycle-related pathways (Fig 8A). Notably, Clusters 1 and 2 exhibited opposite temporal transcriptional trajectories compared to Cluster 4 at O + 1. Clusters 1 and 2 showed high transcriptional activity during Phase A, followed by suppression at O + 1, whereas Cluster 4 displayed the opposite pattern, with relatively low expression during Phase A and marked upregulation at O + 1.

At O + 8, Clusters 2, 3, 4, and 6 showed pronounced downregulation, whereas Cluster 5 (mainly enriched in cofactor biosynthesis, the pentose phosphate pathway, and D-amino acid metabolism) was upregulated. By O + 11, Clusters 1, 2, 3, and 6 exhibited varying degrees of transcriptional recovery. In contrast, Cluster 5 showed decreased expression at this phase.

Temporal clustering analysis of *Gardnerella vaginalis* and non-*L. crispatus* lactobacilli transcripts revealed that *G. vaginalis* exhibited generally high transcriptional expression levels on the third day after menstruation (M + 3), was markedly suppressed after ovulation (O + 1, O + 2), and showed partial recovery during the mid-to-late luteal phase (O + 8, O + 11) (Fig 7B). In contrast, non-*L. crispatus* lactobacilli displayed a transient upregulation on the third day after menstruation (M + 3), followed by a rapid decline before ovulation (O − 1), a subsequent increase after ovulation (O + 1), and overall suppression during the mid-to-late luteal phase (O + 8, O + 11) (Fig 7C). Unlike the distinct temporal modularity trajectories observed across different transcriptional clusters of *Lactobacillus crispatus*, the expression trajectories of *Gardnerella vaginalis* and non-*L. crispatus* lactobacilli were generally consistent. Their KEGG functional enrichment profiles also lacked the cluster-specific functional characteristics exhibited by *Lactobacillus crispatus* (Fig 8B, Fig 8C).

## 5. Discussion

This study demonstrates that although 16S rRNA sequencing indicates a stable composition of the vaginal microbiome across different phases in participant E, metatranscriptomic analysis reveals that *Lactobacillus crispatus* adapts to changes in the host vaginal microenvironment through dynamic transcriptional reprogramming.

During the follicular phase, *L. crispatus* exhibited high transcriptional expression levels of clusters associated with DNA replication and repair, ribosome biogenesis, and cell wall synthesis (Cluster 1), nucleotide metabolism, amino acid synthesis, and core carbon metabolism (Cluster 2), as well as cofactor biosynthesis, the pentose phosphate pathway, and D-amino acid metabolism (Cluster 5). During menstruation, endometrial ischemia and necrosis occur, and menstrual blood flows into the vagina. Its neutral pH and abundant heme iron disrupt the original acidic steady-state environment of the vagina, leading to a transient decrease in *Lactobacillus* abundance and a temporary increase in microbial diversity [15–16]. The high transcriptional activity related to replication, repair, and biosynthesis during the follicular phase reflects the rapid population growth and functional recovery of *L. crispatus* after menstruation.

In contrast to the follicular phase, on the first day after ovulation, the expression of clusters related to basal biosynthesis and central carbon metabolism in *L. crispatus* were suppressed, while clusters associated with carbohydrate transport and utilization, the phosphotransferase system, starch and sucrose metabolism, and galactose metabolism (Cluster 4), nicotinate metabolism and redox-related processes (Cluster 6), and quorum sensing (Cluster 3) were significantly upregulated. Before ovulation, higher estrogen levels promote the accumulation of glycogen in the vaginal epithelium [17]. After ovulation, the effect of progesterone strengthens, and the shedding of superficial epithelial cells increases, releasing the glycogen-rich epithelial cells accumulated before ovulation into the vaginal lumen [18–19]. Therefore, the upregulation of pathways related to carbohydrate transport and utilization on the day after ovulation may reflect a rapid response of *L. crispatus* to changes in available carbohydrate substrates. During the progesterone-dominated luteal phase, cervical mucus viscosity increases and becomes thicker, which may restrict nutrient diffusion and bacterial migration. Concurrently, progesterone promotes the recruitment of neutrophils to the vaginal lumen and enhances their bactericidal activity by activating crosstalk between cervical resident macrophages and neutrophils. Neutrophils release reactive oxygen species while clearing pathogens, inducing local oxidative stress, which may non-specifically inhibit the rapid growth of commensal bacterial communities, thereby reducing the capacity of the vaginal environment to support rapid bacterial proliferation [20,21]. Against this background, the transcriptional activity of *L. crispatus* gradually shifts from growth-related processes to adaptive functions such as substrate acquisition, alternative carbon source utilization, redox homeostasis maintenance, and population coordination.

On the eighth day after ovulation (O + 8), multiple clusters related to basal biosynthesis, core carbon metabolism, and the cell cycle in *Lactobacillus crispatus* showed an overall downward trend, whereas transcript expression clusters associated with cofactor biosynthesis, the pentose phosphate pathway, and D-amino acid metabolism were upregulated. During the mid-to-late luteal phase, under the dominance of progesterone, vaginal epithelial proliferation slows down, glycogen replenishment decreases, and oxidative products accumulate [22,23]. In this context, the coordinated upregulation of the aforementioned pathways may be associated with the adaptive mechanisms of *Lactobacillus crispatus*. For example, upregulation of pentose phosphate pathway transcripts enhances cellular reducing power by generating NADPH [24]; upregulation of transcripts involved in cofactor and riboflavin-derived coenzyme (FMN, FAD) biosynthesis may help maintain redox balance and metabolic activity [25]; and upregulation of D-amino acid metabolism-related transcripts may participate in maintaining cell wall homeostasis [26].

Interestingly, unlike trends observed in other non-*L. crispatus* lactobacilli, at day 11 post-ovulation (O + 11), certain clusters in *L. crispatus* showed upregulation (Clusters 1, 2, 3, and 6), including those associated with genetic information processing, metabolism, and quorum sensing, whereas cluster 4 (carbohydrate transport and utilization, phosphotransferase system, starch and sucrose metabolism, and galactose metabolism) and cluster 5 (cofactor biosynthesis, pentose phosphate pathway, and D-amino acid metabolism) decreased relative to O + 8. These expression characteristics suggest that *L. crispatus* employs a transcriptional regulatory strategy distinct from that of other *Lactobacillus* species during the pre-menstrual phase, which may be associated with alterations in hormone levels. Consistently, a study by Muscò et al. demonstrated that *L. crispatus* exhibits a unique and robust transcriptomic response to sex hormones (a combination of drospirenone and ethinylestradiol). Upon hormonal stimulation, transcripts involved in cell wall synthesis, metabolism, and riboflavin biosynthesis were upregulated, whereas other vaginal lactobacilli (*L. gasseri*, *L. jensenii*, and *L. iners*) exhibited only weak responses or predominantly downregulated transcript expression [27].Currently, the underlying drivers of this expression pattern remain unclear, and further studies incorporating host and microenvironmental data are required to elucidate the molecular mechanisms.

Species-level metatranscriptomic analysis further revealed that non-dominant bacteria did not follow the same transcriptional trajectory as *L. crispatus*. During the transition from the follicular phase to the early luteal phase, the transcriptional levels of transcripts related to central carbon metabolism, biosynthesis, host interaction, and virulence pathways in *Gardnerella vaginalis* and *Fannyhessea vaginae* were downregulated overall. This phenomenon may be related to *L. crispatus* enhancing carbohydrate substrate acquisition in the glycogen-rich environment of the early luteal phase, producing more lactic acid and maintaining a lower pH, thereby ecologically restricting *G. vaginalis* and other opportunistic bacteria [28–30]. Meanwhile, non-*L. crispatus* lactobacilli also showed transcriptional activity across all clusters on the day after ovulation, possibly due to enhanced utilization of carbohydrate substrates during the glycogen-rich phase. In contrast to *L. crispatus*, non-*L. crispatus* lactobacilli showed downregulation of transcripts related to carbon and energy metabolism during the mid-to-late luteal phase, which may reflect a comparatively lower capacity to maintain metabolic activity under conditions of reduced glycogen and other carbon source availability in the vaginal environment. This interpretation is supported by the observations of Navarro et al., who demonstrated that the growth of non-*L. crispatus* lactobacilli species is more strongly affected by glycogen limitation than that of *L. crispatus* [31]. The distinct transcriptional trends between *L. crispatus* and non-*L. crispatus* species indicate that the *Lactobacillus* genus is not a functionally homogeneous group, and the functional heterogeneity among different species may be an important reason explaining why *L. crispatus* is often associated with a more stable and protective vaginal ecosystem [32]. In contrast, *G. vaginalis* exhibited a general upregulation of transcripts related to carbon metabolism, microbial metabolism in diverse environments, and secondary metabolite biosynthesis during the mid-to-late luteal phase, suggesting a time window when the proliferative advantage of *L. crispatus* weakens and niche constraints relatively loosen, allowing *G. vaginalis* to achieve opportunistic recovery using alternative metabolic strategies. This observation aligns with previous findings regarding the high metabolic plasticity of *G. vaginalis* [33]. Although *G. vaginalis* and *F. vaginae* were present at low relative abundance, the detection of differentially expressed transcripts suggests that low-abundance taxa may remain transcriptionally active and contribute to vaginal microecological interactions through phase-specific regulation of metabolic and virulence-related pathways [34].

Several limitations should be noted when interpreting the results of this study. Firstly, this longitudinal multi-omics analysis was conducted on a single individual. Although this design allows high-resolution characterization of the functional dynamics of *Lactobacillus crispatus* across different phases of the menstrual cycle, inter-individual heterogeneity remains. Therefore, larger population cohorts are required to verify the generalizability of the dynamic patterns observed in this study. Secondly, menstrual samples were not included in the present study. Menstrual specimens generally contain abundant host-derived RNA, blood components, and exfoliated epithelial cells. These factors may interfere with microbial transcriptional signals and reduce the stability and interpretability of functional comparisons across different time points; however, the absence of menstrual samples also precludes our ability to assess the transcriptional response characteristics of *L. crispatus* during this critical time point of menstruation. Finally, future in vitro co-culture experiments using vaginal epithelial cells can be performed to further explore the potential mechanisms by which hormonal fluctuations regulate the transcriptional dynamics of *Lactobacillus crispatus*.

## 6. Conclusions

Although the dominance of *Lactobacillus crispatus* in the CST-I vaginal microbiome remains stable across the non-menstrual phases at the taxonomic level, its functional activity undergoes pronounced phase-specific transcriptional reprogramming. *L. crispatus* dynamically adjusts pathways related to growth, carbohydrate utilization, stress response, and metabolism to adapt to changing vaginal microenvironments. This metabolic plasticity and rhythmic functional remodeling likely represent key mechanisms underlying its long-term persistence and ecological dominance in healthy vaginal communities.

## Supporting information

S1 Table Metatranscriptomic gene-level functional annotation and expression matrix. (XLSX)
